# Supplementary material for: Contextuality without nonlocality in a superconducting quantum system
Source: Nat Commun. 2016 Oct 4;7:12930. doi: 10.1038/ncomms12930 (PMC5059491; doi:10.1038/ncomms12930)
Supplement: Supplementary Information — Supplementary Figure 1, Supplementary Note 1 and Supplementary References. [file ncomms12930-s1.pdf]

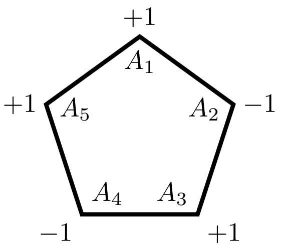

Supplementary Figure 1: The KCBS pentagram, with a specific preassigned set of measurement outcomes that maximize the winning probability

Supplementary Note 1. The KCBS noncontextuality test as a hypothesis test

In this appendix the P-value analysis is discussed. First, P-values in general and hypothesis tests are briefly discussed. Second, the experiment performed is phrased informally as a game, which will turn out to be convenient for the calculation of a P-value. Third, the notation and definitions are written down formally. Fourth, the properties of an ϵ-bounded i.i.d. noncontextual hidden variable (NCHV) model are explicitly formulated. Fifth, the probability for any ϵ-bounded i.i.d. NCHV model to win the game is calculated, which is needed for the P-value calculation. Sixth, a method to confidently upper bound the amount of incompatibility ϵ is detailed. Finally, the analysis is directly applied to the experiment performed to obtain a P-value.

Tests of any HV model can be phrased as a hypothesis test, in which one aims to reject the null hypothesis that an underlying HV model explains the data. The P-value is the maximum probability that *if* the null hypothesis were to hold, the data would have been *at least* as extreme as observed. A low P-value suggests then that the null hypothesis should be rejected. For the experiment performed, the null hypothesis is that the experiment was governed by an ϵ-bounded i.i.d. NCHV model (formally defined below), and the P-value is the maximum probability that *any* ϵ-bounded i.i.d. NCHV model could have produced data at least as extreme as observed.

a KCBS inequality as a win/lose game

To find an upper bound on the P-value, the KCBS inequality is phrased as a win/lose game [1]. The P-value can then be expressed as the maximum probability that any ϵ-bounded i.i.d. NCHV model would have produced *at least* as many wins as observed.

$$P\text{-value} = \max_{\substack{\epsilon\text{-bounded} \\ \text{i.i.d. NCHV}}} \Pr\left[\#\text{ of wins at least as large as observed} \mid \epsilon\text{-bounded i.i.d. NCHV}\right] . \tag{1}$$

If phrased correctly, any ϵ-bounded i.i.d. NCHV model will have a certain maximum winning probability which is strictly smaller than the maximum winning probability of a contextual model, where in particular β_{win} is the maximum winning probability over all ϵ-bounded i.i.d. NCHV models [1].

$$P\text{-value} \leq \sum_{i=c}^{n} \binom{n}{i} \left(\beta_{\text{win}}\right)^{i} \left(1-\beta_{\text{win}}\right)^{n-i} . \tag{2}$$

The KCBS inequality can be violated by minimizing the correlators of the products of outcomes. This corresponds to a game where a random context is selected, after which the game is won when the two observed outcomes are different.

b Notation and definitions

The experiment consists of n trials, where in a specific trial $1 \leq l \leq n$ first two ordered measurements are selected. The two measurements have corresponding random variables $\#1^l$ (measured first) and $\#2^l$ (measured second) with outcomes i and j, where the possible ordered combinations of (i,j) are restricted to be in the set $\mathcal{D} = \{(1,2),(3,2),(3,4),(5,4),(5,1)\}$. The restriction of the possible ordered contexts to the set $\mathcal{D}$ is due to the fact that the extended KCBS inequality is phrased entirely with these ordered contexts. Secondly, the outcomes of the two measurements are recorded as a_i^l, a_j^l with corresponding random variables A_i^l and A_j^l. The score for a trial is $c^l = \delta(a_i^l + a_j^l) \in \{0,1\}$, i.e. for the win/lose game described a trial is won when $c^l = 1$, or equivalently, $a_i^l \neq a_j^l$. The total amount of wins c then equals $c = \sum_{l=1}^{n} c^l$. The random variable H^l includes all previous instances of hidden variables h and the history of all previous in- and outputs $\left(i^l, j^l, a_i^l, a_j^l\right)_{k=1}^{l-1}$.

c Formulating the null hypothesis

The null hypothesis that we are testing here is that the experiment is governed by an ϵ-bounded i.i.d. noncontextual hidden variable model, which we will define below. A hidden-variable model by itself means that, if $\Pr(A_i^l = a_i^l, A_j^l = a_j^l \mid \#1 = i, \#2 = j)$ is the probability of observing outcomes a_i^l and a_j^l during the l-th trial of the experiment in which we first measure i and then j, then this probability can be expressed as

$$\Pr(A_i^l = a_i^l, A_j^l = a_j^l \mid \#1 = i, \#2 = j) = \int \mathrm{d}\mu(h) \Pr(A_i^l = a_i^l, A_j^l = a_j^l \mid \#1 = i, \#2 = j, h) \;, \tag{3}$$

where $\mathrm{d}\mu$ denotes some probability measure over hidden variables h.
Typically, one would make the following assumptions.

1(a). ***Uniform randomness***. Conditioned on a given value h of the hidden variable, the probability of selecting an ordered context $(i, j) \in \mathcal{D}$ in each trial l is uniform

$$\forall\ (i,j) \in \mathcal{D}, \forall\ l,\ \ \Pr(\#1^l = i^l,\ \#1^l = j^l, h) = \frac{1}{5} \;. \tag{4}$$

In the actual performed experiment the measurements were measured in a predetermined fashion. The uniform randomness criteria must then be augmented by

1(b). ***Independent and identically distributed (i.i.d)***. For any context (i, j) the outcomes of the experiment during each trial are independent of the history of the experiment

$$\forall (i,j,l),\ A_i^l \perp\!\!\!\perp H^l,\ A_j^l \perp\!\!\!\perp H^l \;, \tag{5}$$

and have equal probability distributions for all l. This is equivalent to saying that the distribution over hidden variables determining the outcomes is the same in every trial.

2. ***Sequentiality of the measurements***. Each of the measurements are separated and ordered in time, so that causality prevents signalling from any of the future attempts to current or previous attempts. In particular, this also implies that if h is the hidden variable of the present trial

$$\Pr(A_i = a_i, A_j = a_j | \#1 = i, \#2 = j, h) = \Pr(A_i = a_i | \#1 = i, h) \Pr(A_j = a_j | \#1 = i, \#2 = j, h) \;, \tag{6}$$

since the outcome of the first measurement cannot depend on whether or not a measurement will be performed in the future.

3. ***Bounded incompatibility/noncontextuality***. The incompatibility is ϵ-bounded [2], in the sense that for any instance h of the hidden variable and any context (i, j), there exists $\epsilon_{(i,j),h}$ such that[1]

$$-\epsilon_{(i,j),h} \leq \Pr(A_j = a_j | \#1 = j, h) - \Pr(A_j = a_j | \#1 = i, \#2 = j, h) \leq \epsilon_{(i,j),h} \;, \tag{7}$$

with $\epsilon_{(i,j),h} \in \{0, 1\}$, and hence

$$-\epsilon_{(i,j)} \leq \Pr(A_j = a_j | \#1 = j) - \Pr(A_j = a_j | \#1 = i, \#2 = j) \leq \epsilon_{(i,j)} \;, \tag{8}$$

for $\epsilon_{(i,j)} := \int \mathrm{d}\mu(h) \epsilon_{(i,j),h}$ being the average taken over the hidden variables such that the *average incompatibility* is ϵ-bounded,

$$-\epsilon \leq \frac{1}{5} \int \mathrm{d}\mu(h) \sum_{(i,j) \in \mathcal{D}} \epsilon_{(i,j),h} = \frac{1}{5} \sum_{(i,j) \in \mathcal{D}} \epsilon_{(i,j)} \leq \epsilon \;. \tag{9}$$

What we will test here is the null hypothesis of a hidden variable model satisfying (1a), (1b), (2) and (3), which we will call an ϵ-bounded i.i.d. NCHV model. We note that the analysis of [1] allows arbitrary memory for the hidden-variable model, that is, the i.i.d. assumption is not needed. However, in the present experiment this assumption is necessary.

[1]Note the lack of a factor of a half which is standard in the literature.

d Upper bounding β_{win}

In this section the maximum winning probability β_{win} is upper bounded for an ϵ-bounded i.i.d. NCHV model as specified above.

$$\begin{aligned}
\beta_{\text{win}} &= \int \mathrm{d}\mu(h) \sum_{(i,j)\in\mathcal{D}} \Pr(\#1=i,\#2=j,h)\Pr(\text{win}|\#1=i,\#2=j,h) && (10)\\
&= \frac{1}{5}\int \mathrm{d}\mu(h) \sum_{(i,j)\in\mathcal{D}} \Pr(\text{win}|\#1=i,\#2=j) && (11)\\
&= \frac{1}{5}\int \mathrm{d}\mu(h) \sum_{(i,j)\in\mathcal{D}} \sum_{a\in\{-1,1\}} \Pr(A_i=a,A_j=-a|\#1=i,\#2=j,h) && (12)\\
&= \frac{1}{5}\int \mathrm{d}\mu(h) \sum_{(i,j)\in\mathcal{D}} \sum_{a\in\{-1,1\}} \Pr(A_i=a|\#1=i,h)\Pr(A_j=-a|\#1=i,\#2=j,h) && (13)\\
&= \frac{1}{5}\int \mathrm{d}\mu(h) \sum_{(i,j)\in\mathcal{D}} \Pr(A_i=1|\#1=i,h)\Pr(A_j=-1|\#1=i,\#2=j,h)\\
&\qquad + \Pr(A_i=-1|\#1=i,h)\Pr(A_j=1|\#1=i,\#2=j,h) && (14)\\
&= \frac{1}{5}\int \mathrm{d}\mu(h) \sum_{(i,j)\in\mathcal{D}} \Pr(A_i=1|\#1=i,h)\left(1-\Pr(A_j=1|\#1=i,\#2=j,h)\right)\\
&\qquad + \left(1-\Pr(A_i=1|\#1=i,h)\right)\Pr(A_j=1|\#1=i,\#2=j,h) && (15)\\
&= \frac{1}{5}\int \mathrm{d}\mu(h) \sum_{(i,j)\in\mathcal{D}} \Pr(A_i=1|\#1=i,h)\\
&\qquad + \left(1-2\Pr(A_i=1|\#1=i,h)\right)\Pr(A_j=1|\#1=i,\#2=j,h) && (16)\\
&\le \frac{1}{5}\int \mathrm{d}\mu(h) \sum_{(i,j)\in\mathcal{D}} \Pr(A_i=1|\#1=i,h)\\
&\qquad + \left(1-2\Pr(A_i=1|\#1=i,h)\right)\Pr(A_j=1|\#1=j,h)+\epsilon_{(i,j),h} && (17)\\
&\le \epsilon + \frac{1}{5}\int \mathrm{d}\mu(h) \sum_{(i,j)\in\mathcal{D}} \Pr(A_i=1|\#1=i,h)\\
&\qquad + \left(1-2\Pr(A_i=1|\#1=i,h)\right)\Pr(A_j=1|\#1=j,h) && (18)
\end{aligned}$$

where in equation (10) the winning probability is written as an integral over hidden variables h, and a conditional sum over selecting measurements i and j. Equation (11) follows from $\Pr(\#1=i,\#2=j|h)=\frac{1}{5},\ \forall(i,j)\in\mathcal{D},\forall h$. In the case of the present experiment, we rely on condition (1b). That is, there is no random choice *a priori*, but instead such random selection is simulated after the fact.

In equation (12) the winning condition is formulated in the summands for a fixed $a\in\{-1,1\}$. Equation (13) is based on the fact that the outcome of A_i cannot depend on #2 since it hasn't been measured yet. In equation (14) the terms depending on $a\in\{-1,1\}$ are written out explicitly. Equations (15) and (16) follow from $\Pr(A_i=1|\#1=i)=(1-\Pr(A_i=-1|\#1=i))$ and rewriting. The inequalities in equations (17) and (18) follow from the inequalities

$$-\epsilon_{(i,j),h} \le \Pr(A_j=a_j|\#1=j,h)-\Pr(A_j=a_j|\#1=i,\#2=j,h) \le \epsilon_{(i,j),h}\ , \quad (19)$$

$$\frac{1}{5}\int \mathrm{d}\mu(h) \sum_{(i,j)\in\mathcal{D}} \epsilon_{(i,j),h} \le \epsilon\ . \quad (20)$$

Note that the values $\epsilon_{(i,j),h}$ cannot be known, but fortunately ϵ, the parameter of relevance, *can* be bounded

from above as will be shown in the next section. The integral term equals

$$\int \mathrm{d}\mu(h) \sum_{(i,j)\in\mathcal{D}} \Pr(A_i = 1|\#1 = i, h) + (1 - 2\Pr(A_i = 1|\#1 = i, h)) \Pr(A_j = 1|\#1 = j, h) \tag{21}$$

$$= \int \mathrm{d}\mu(h) \sum_{(i,j)\in\mathcal{D}} \Pr(A_i = 1|\#1 = i, h) + \Pr(A_j = 1|\#1 = j, h)$$

$$- 2\Pr(A_i = 1|\#1 = i) \Pr(A_j = 1|\#1 = j, h) \tag{22}$$

$$= 2\int \mathrm{d}\mu(h) \left(\sum_{i=1}^{5} \Pr(A_i = 1|\#1 = i, h) - \sum_{(i,j)\in\mathcal{D}} \Pr(A_i = 1|\#1 = i, h) \Pr(A_j = 1|\#1 = j, h) \right) \tag{23}$$

$$\leq 4 \ , \tag{24}$$

where equation (22) follows from rewriting and equation (23) follows from the fact that

$$\sum_{(i,j)\in\mathcal{D}} \Pr(A_i = 1|\#1 = i, h) + \Pr(A_j = 1|\#1 = j, h) = 2\sum_{i=1}^{5} \Pr(A_i = 1|\#1 = i, h) \ . \tag{25}$$

The integral term achieves its maximum when all the probability mass $\mathrm{d}\mu(h)$ is concentrated on the deterministic distributions (i.e. $\forall i,\ \Pr(A_i = 1|\#1 = i, h) \in \{0, 1\}$) that maximize equation (23). One can easily see that the best strategy is to alternate the outcomes as in Figure 1. For these distributions, the sum achieves its maximum value of 2, from which the upper bound in equation (24) follows. Combining equations (24) and (18) yields

$$\beta_{\text{win}} \leq \frac{4}{5} + \epsilon \ . \tag{26}$$

e Upper bounding the incompatibility ϵ

If the assumption is made that the experiment behaves in an i.i.d fashion, then the average incompatibility ϵ is a parameter that can be estimated by performing a game separate from the main experiment. Informally, the game is played over n trials, where a total score g^n_{avg} is assigned for the whole game. By construction, the absolute value of the expectation value of the score (i.e. $\left|\mathbb{E}[G^n_{\text{avg}}]\right|$) will be an estimate for ϵ.

However, since there are only a finite amount of trials the observed score will deviate from its expectation value, which is the quantity of interest. As will be shown, the maximum deviation t between the observed score g^n_{avg} and its expectation value $\mathbb{E}[G^n_{\text{avg}}]$ can be bounded with high probability. This allows for the ϵ parameter to be upper bounded with high probability by the absolute value of the observed score (i.e. $\left|g^n_{\text{avg}}\right|$), plus some error margin t.

More formally, a game is played consisting of n trials, where in each trial l an ordered context $(i, j) \in \mathcal{D}$ is uniformly selected. Then either (i, j) or (j, i) is measured, depending on the outcome of a uniform random variable X^l taking values $x^l = 1$ or $x^l = -1$, respectively. The outcome a^l_j corresponding to the random variable A^l_j is recorded. The concrete score g^l, corresponding to the random variable G^l, is equal to 2 when $x^l = 1,\ a^l_j = 1$, equal to -2 when $x^l = -1,\ a^l_j = 1$ and 0 for all other instances. The average score at the end of n trials equals

$$g^n_{\text{avg}} = \frac{1}{n}\sum_{l=1}^{n} g^l \tag{27}$$

with the associated random variable G^n_{avg}. The expectation value of G^n_{avg} taken over all possible contexts

$(i, j) \in \mathcal{D}$ and values of X^l satisfies

$$\mathbb{E}\left[G_{\text{avg}}^n\right] = \frac{1}{5} \sum_{(i,j)\in\mathcal{D}} \left(\Pr(A_j = 1|\#1 = j) - \Pr(A_j = 1|\#1 = i, \#2 = j)\right) \ . \tag{28}$$

From equations (9) and (28) ϵ can be estimated by $\left|\mathbb{E}[G_{\text{avg}}^n]\right|$, i.e. the absolute value of the expectation value of the score over n trials. Since there is only a finite amount of samples $\mathbb{E}[G_{\text{avg}}^n]$ cannot be estimated perfectly, so that for any experiment the observed score g_{avg}^n will deviate from the expectation value $\mathbb{E}[G_{\text{avg}}^n]$. That is, $g_{\text{avg}}^n - t \leq \mathbb{E}[G_{\text{avg}}^n] \leq g_{\text{avg}}^n + t$, or equivalently, $\left|g_{\text{avg}}^n - \mathbb{E}[G_{\text{avg}}^n]\right| \leq t$ for some $t > 0$. The probability that the observed average value corresponding to a sequence of i.i.d. random variables (the average score g_{avg}^n in the present case) deviates at most t from the expectation value of the average of those random variables ($\mathbb{E}[G_{\text{avg}}^n]$ in the present case) can be upper bounded with Bentkus' inequality [3]. Before stating Bentkus' inequality, we define

$$P_{n,k}\left(\mathbb{B}_\gamma\right) := \sum_{i=k}^{n} \binom{n}{i} \gamma^i \left(1-\gamma\right)^{n-i} \tag{29}$$

and

$$\mathring{P}_{n,y}\left(\mathbb{B}_\gamma\right) = \left(P_{n,\lfloor y\rfloor}\left(\mathbb{B}_\gamma\right)\right)^{1-(y-\lfloor y\rfloor)} \left(P_{n,\lceil y\rceil}\left(\mathbb{B}_\gamma\right)\right)^{y-\lfloor y\rfloor} \ . \tag{30}$$

Theorem 1 (Bentkus' inequality)**.** *Let $M^1, M^2, \dots, M^n$ be a martingale sequence with differences $X^l = M^l - M^{l-1}$ and $M^0 = 0$. If for $l = 1 \dots n$ the differences satisfy the following boundedness condition,*

$$\Pr(-\alpha^l \leq X^l \leq 1 - \alpha^l) = 1 \ , \tag{31}$$

then

$$\Pr(M^n \geq t) \leq e\mathring{P}_{n,t+n\gamma}\left(\mathbb{B}_\gamma\right) \tag{32}$$

with $\gamma = \sum_{i=1}^n \alpha_i/n$.

As we show in the corollary below, Bentkus' inequality can be phrased in a way to get a bound on $\Pr\left(\left|g_{\text{avg}}^n - \mathbb{E}[G_{\text{avg}}^n]\right| \geq t\right)$.

Corollary 1. *Let $X_1, X_2, \dots, X_n$ be a sequence of i.i.d. variables satisfying $-a \leq X_i \leq a$, $\forall i \in \{1, 2, \dots, n\}$. Define $\overline{X} = \frac{1}{n}\sum_{i=1}^n X^i$, then*

$$\Pr\left(\left|\overline{X} - \mathbb{E}[\overline{X}]\right| \geq t\right) \leq 2e\mathring{P}_{n,n\left(\frac{t+2a}{4a}\right)}\left(\mathbb{B}_{1/2}\right) \ . \tag{33}$$

Proof. Define the sequence $M^1, M^2, \dots, M^n$ with $M^l = \frac{1}{4a}\sum_{i=1}^l \left(X^i - \mathbb{E}\left[\overline{X}\right]\right)$. This is a martingale sequence with $\mathbb{E}[M^{l+1}|M^1, M^2, \dots, M^l] = 0$ since the sequence $X^1, X^2, \dots, X^n$ is i.i.d., and has bounded differences $-\frac{1}{2} \leq M^l - M^{l-1} = \frac{1}{4a}\left(X^l - \mathbb{E}[\overline{X}]\right) \leq \frac{1}{2}$ since $-a \leq X^l \leq a$. Bentkus' inequality can then be applied to the sequence $M^1, M^2, \dots, M^n$ with $\gamma = \sum_{i=1}^n \alpha/n = 1/2$,

$$\Pr\left(M^n \geq \frac{nt}{4a}\right) \leq e\mathring{P}_{n,\frac{nt}{4a}+\frac{n}{2}}\left(\mathbb{B}_{1/2}\right) = e\mathring{P}_{n,n\left(\frac{t+2a}{4a}\right)}\left(\mathbb{B}_{1/2}\right) \ . \tag{34}$$

Rewriting yields

$$\Pr\left(M^n \geq \frac{nt}{4a}\right) = \Pr\left(\frac{1}{4a}\sum_{i=1}^{n}\left(X_i - \mathbb{E}[\overline{X}]\right) \geq \frac{nt}{4a}\right) \tag{35}$$

$$= \Pr\left(\sum_{i=1}^{n}\left(X_i - \mathbb{E}[\overline{X}]\right) \geq nt\right) \tag{36}$$

$$= \Pr\left(n\left(\overline{X} - \mathbb{E}[\overline{X}]\right) \geq nt\right) \tag{37}$$

$$= \Pr\left(\overline{X} - \mathbb{E}[\overline{X}] \geq t\right) \ , \tag{38}$$

so that

$$\Pr\left(\overline{X} - \mathbb{E}[\overline{X}] \geq t\right) \leq e\mathring{P}_{n,n\left(\frac{t+2a}{4a}\right)}\left(\mathbb{B}_{1/2}\right) \ . \tag{39}$$

The same procedure can be followed for the martingale sequence $-M^1, -M^2, \ldots, -M^n$, so that

$$\Pr\left(\left|\overline{X} - \mathbb{E}[\overline{X}]\right| \geq t\right) \leq 2e\mathring{P}_{n,n\left(\frac{t+2a}{4a}\right)}\left(\mathbb{B}_{1/2}\right) \ . \tag{40}$$

□

In other words, with equation (33) it is possible to obtain a *confidence interval*, relating the maximum deviation one would expect to see between the average of i.i.d. variables and their expectation value for a certain probability. In particular, it can be used to bound the probability that $\left|g^n_{\text{avg}} - \mathbb{E}[G^n_{\text{avg}}]\right| \geq t$. Specifically, we apply equation (33) to the sequence of i.i.d random variables $G^1, G^2, \ldots, G^n$, where the mean over the random variables is $\frac{1}{n}\sum_{l=1}^{n} G^l = G^n_{\text{avg}}$, and $-2 \leq G^l \leq 2$ so that $a = 2$. Equation (33) then yields

$$\Pr\left(\left|G^n_{\text{avg}} - \mathbb{E}[G^n_{\text{avg}}]\right| \geq t\right) \leq 2e\mathring{P}_{n,n\left(\frac{t+4}{8}\right)}\left(\mathbb{B}_{1/2}\right) \ . \tag{41}$$

In particular, equation (41) can be applied to the observed score g^n_{avg}, which allows for a parameter estimation of ϵ with some confidence interval t,

$$\Pr\left(\left|g^n_{\text{avg}} - \mathbb{E}[G^n_{\text{avg}}]\right| \geq t\right) \leq 2e\mathring{P}_{n,n\left(\frac{t+4}{8}\right)}\left(\mathbb{B}_{1/2}\right) \tag{42}$$

That is, with probability less than $2e\mathring{P}_{n,n\left(\frac{t+4}{8}\right)}\left(\mathbb{B}_{1/2}\right)$, the observed data g^n_{avg} will satisfy $\left|g^n_{\text{avg}} - \mathbb{E}[G^n_{\text{avg}}]\right| \geq t$. Using equations (9) and (28), ϵ can then be upper bounded by $\left|g^n_{\text{avg}}\right| + t$ with probability greater than $1 - 2e\mathring{P}_{n,n\left(\frac{t+4}{8}\right)}(\mathbb{B}_{1/2})$. We note that we are free to choose t and n independent of recorded data to aim for a bound on the probability that is good enough to confidently upper bound ϵ by $\left|g^n_{\text{avg}}\right| + t$. Setting $t = 0.005$ with $n = 9207101$ measurements (which constitutes the first half of the data collected), we find

$$\Pr\left(\left|g^n_{\text{avg}} - \mathbb{E}[G^n_{\text{avg}}]\right| \geq t\right) \leq 2e\mathring{P}_{n,n\left(\frac{t+4}{8}\right)}\left(\mathbb{B}_{1/2}\right) \tag{43}$$

$$\leq 4.1 \cdot 10^{-4} \ . \tag{44}$$

f Statistical analysis

The analysis above can then directly be applied to the performed contextuality experiment. The total analysis consists of the estimation of ϵ and the testing of the null hypothesis as formulated above. These two tests are performed on two disjoint parts of the data. Recall that we take it as a given that the NCHV model is i.i.d., that is, the devices perform the same in every single trial. This allows us to make separated estimates.

Before proceeding, we clarify that the contexts were not chosen randomly during the course of the experiment, but note that making random measurements is not necessary when testing an i.i.d. model. Furthermore, the P-value analysis of [1] we use here assumes that the number of trials n is selected independently of the data. Here, we had to make an explicit assumption that we were able to pick n uninfluenced by an HV model, since the data had already been taken. We again note that the model to be tested is i.i.d., and also that it has no memory. We emphasize that no such assumptions were made in the analysis of the recent loophole-free Bell test [4], where in particular the model was allowed to have full memory.

As shown in the previous section, with probability less than $4.1 \cdot 10^{-4}$ the condition $\epsilon \leq \left|g^n_{\text{avg}}\right| + 0.005$ will hold. Calculating g^n_{avg} from recorded data we find $\left|g^n_{\text{avg}}\right| \leq 0.036286$, so that $\beta_{\text{win}} \leq \frac{4}{5} + \left|g^n_{\text{avg}}\right| + t = \frac{4}{5} + 0.036286 + 0.005 = 0.841286$.

Second, we can now compute the P-value of testing an ϵ-bounded i.i.d. NCHV. We remark that for large n as in the present experiment, the distribution is approximately normal, meaning that an estimate of the P-value based on standard deviations is approximately accurate. However, the analysis of [1] gives a *tight* bound on the P-value. This can be calculated using the results of [1] by setting $\beta_{\text{win}} = \frac{4}{5} + \left|g^n_{\text{avg}}\right| + t = 0.841286$, counting the amount of wins c and trials n and applying equation (2). We have $c = 3912769$, and $n = 4603450$, yielding

$$P\text{-value} \leq \sum_{i=c}^{n} \binom{n}{i} (\beta_{\text{win}})^i (1-\beta_{\text{win}})^{n-i} \tag{45}$$

$$\leq 2.96 \cdot 10^{-575} \; . \tag{46}$$

Supplementary References

[1] Elkouss, D. & Wehner, S. (Nearly) optimal P-values for all Bell inequalities. Preprint at http://arxiv.org/abs/1510.07233 (2015).

[2] Gühne, O.*et al.* Compatibility and noncontextuality for sequential measurements. *Phys. Rev. A* **81**, 022121 (2010).

[3] Bentkus, V. On hoeffding's inequalities, *Annals of Probability*, 1650-1673 (2004).

[4] Hensen, B. *et al.* Loophole-free bell inequality violation using electron spins separated by 1.3 kilometres. *Nature* **526**, 682-686 (2015).
